# Recent Progress of Activity-Based Fluorescent Probes for Imaging Leucine Aminopeptidase

**DOI:** 10.3390/bios13070752

**Published:** 2023-07-21

**Authors:** Ze-Jun Li, Cai-Yun Wang, Liang Xu, Zhen-Yu Zhang, Ying-Hao Tang, Tian-Yi Qin, Ya-Long Wang

**Affiliations:** 1 Key Laboratory of Biomedical Engineering of Hainan Province, School of Biomedical Engineering, Hainan University, Haikou 570228, China; lizejun@hainanu.edu.cn (Z.-J.L.); ycai@hainanu.edu.cn (C.-Y.W.); lxu@hainanu.edu.cn (L.X.); zyzhang@hainanu.edu.cn (Z.-Y.Z.); yhtang@hainanu.edu.cn (Y.-H.T.); 2 One Health Institute, Hainan University, Haikou 570228, China

**Keywords:** leucine aminopeptidase (LAP), fluorescence probe, fluorescence imaging

## Abstract

Leucine aminopeptidase (LAP) is an important protease that can specifically hydrolyze Leucine residues. LAP occurs in microorganisms, plants, animals, and humans and is involved in a variety of physiological processes in the human body. In the physiological system, abnormal levels of LAP are associated with a variety of diseases and pathological processes, such as cancer and drug-induced liver injury; thus, LAP was chosen as the early biochemical marker for many physiological processes, including cancer. Considering the importance of LAP in physiological and pathological processes, it is critical that high-efficiency and dependable technology be developed to monitor LAP levels. Herein, we summarize the organic small molecule fluorescence/chemiluminescence probes used for LAP detection in recent years, which can image LAP in cancer, drug-induced liver injury (DILI), and bacteria. It can also reveal the role of LAP in tumors and differentiate the serum of cirrhotic, drug-induced liver injury and normal models.

## 1. Introduction

Proteases play an indispensable role in the human body, participating in various physiological processes, such as protein digestion, cell proliferation, and apoptosis [1,2,3,4]. Leucine aminopeptidase (LAP) is an important protease that belongs to the M1 and M17 peptidase families. It can selectively cleave N-terminal leucine residues from substrates and is highly prevalent in microorganisms, plants, animals, and humans [5,6,7,8,9]. Aberrant levels of LAP are associated with several diseases or disorders of pathological progress, such as liver dysfunction, tumor cell proliferation invasion and angiogenesis, pancreatic and bile duct diseases, drug resistance endometrial carcinoma, etc., allowing LAP to serve as the diagnostic or prognostic biomarker of liver injury, epithelial ovarian cancer, and breast cancer in clinical practice [10,11,12,13,14,15,16]. Therefore, monitoring LAP activity is critical to diagnose LAP-related diseases in the early stages. 

Feasible methods for LAP detection, including electrophoretically mediated microanalysis (EMMA), capillary electrophoresis (CE) coupled with electrochemiluminescence (ECL), high-performance liquid chromatography (HPLC), and ultraviolet detection, have been reported [17,18,19]. Although these methods have a good ability to detect LAP, their application is limited to in vitro. Compared with these approaches, fluorescent imaging is the more ideal method for living cells or complicated biological specimens, as they are non-invasive and possess high sensitivity, great selectivity, real-time monitoring and high spatial and temporal resolution. The small molecular fluorescent/chemiluminescent probe, a powerful tool for optical imaging, has attracted a great deal of attention and made significant advancements in recent years [20,21,22,23,24,25,26]. The luminescence mechanism of fluorescent probes includes internal charge transfer (ICT), Förster resonance energy transfer (FRET), aggregation-induced emission (AIE), excited-state intramolecular proton transfer (ESIPT), and other mechanisms.

In this review, we summarize the research progress of organic small-molecule probes for LAP detection and imaging, specifically the chemical structures, design principles, and biological applications of the probe. The purpose of this review is to conclude the latest development of fluorescent probes for detection and imaging of LAP. Finally, we hope that this review can provide a general overview of the research design of LAP probes, inspire researchers to develop more novel LAP probes for bioimaging, and reveal the effect and roles of LAP in the physiological and pathological processes.

## 2. Fluorescent/Chemiluminescent Probes for LAP

### 2.1. Leu Residue Hydrolysis

#### 2.1.1. DCDHF-Based Fluorescence Probes

**Figure 1:**
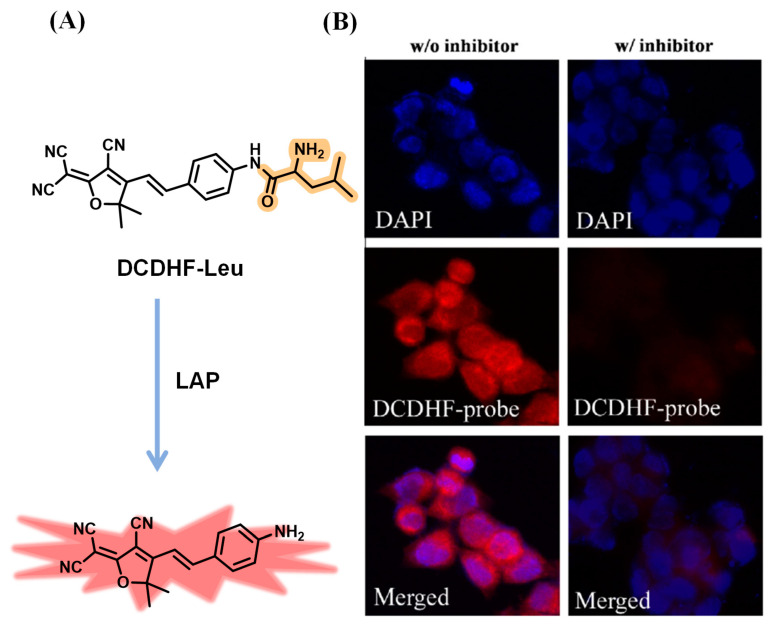
(**A**) Illustration of sensing mechanism of DCDHF-Leu. (**B**) HCT 116 cells imaging with or without bestatin (red: DCDHF-Leu, blue: DAPI). (Reproduced with permission from [27], Copyright 2011, Elsevier).

In 2011, Hong et al. reported a small molecular probe, named DCDHF-Leu, for the detection of LAP (Figure 1A). DCDHF-Leu was constructed by condensing 2-dicyanomethylene-3-cyano-2,5-dihydrofuran (DCDHF) with L-leucine as the recognition group for LAP [27]. DCDHF-Leu exhibited nearly no fluorescence emission in solution. When mixed with LAP, it showed a dramatic fluorescence increase (10-fold) at 605 nm, and the color of the reaction solution changed from yellow to red. Furthermore, DCDHF-Leu was verified for LAP detection in HCT 116 cells. It exhibited obvious red fluorescence in HCT 116 cells. However, in the presence of bestatin, an inhibitor of LAP, it showed a weak fluorescence signal (Figure 1B). 

**Figure 2:**
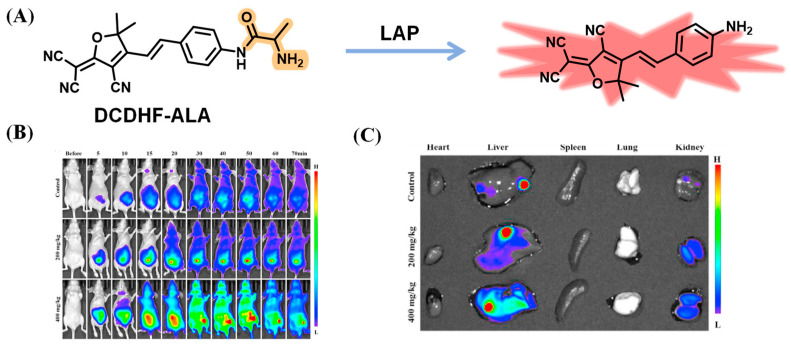
(**A**) Proposed mechanism of DCDHF-Ala. Fluorescence imaging of cancer mouse (**B**) and main organs (**C**) with DCDHF-ALA. (Reproduced with permission from [28], Copyright 2021, Elsevier).

Based on the same electron acceptor, Qin et al. employed alanine (ALA) as a reaction unit for imaging LAP in vivo (Figure 2A) [28]. DCDHF-ALA showed a ratio fluorescence that decreased at 569 nm and increased at 617 nm after the addition of LAP, indicating remarkable specificity to LAP. When DCDHF-ALA was applied in the biological experiment, obvious red fluorescence occurred in A549 and HeLa cells, and the fluorescence intensity gradually enhanced with the extension of the incubation time in zebrafish. The researchers also investigated in vivo imaging of DCDHF-ALA in a tumor mouse. The tumor location was marked with the fluorescence signal after injection with DCDHF-ALA (Figure 2B). Additionally, compared with the other main organs, the liver possessed the strongest luminescence in the liver injury mouse model (Figure 2C). 

#### 2.1.2. Fluorescence Probes Based on Rhodamine and Its Derivates

**Figure 3:**
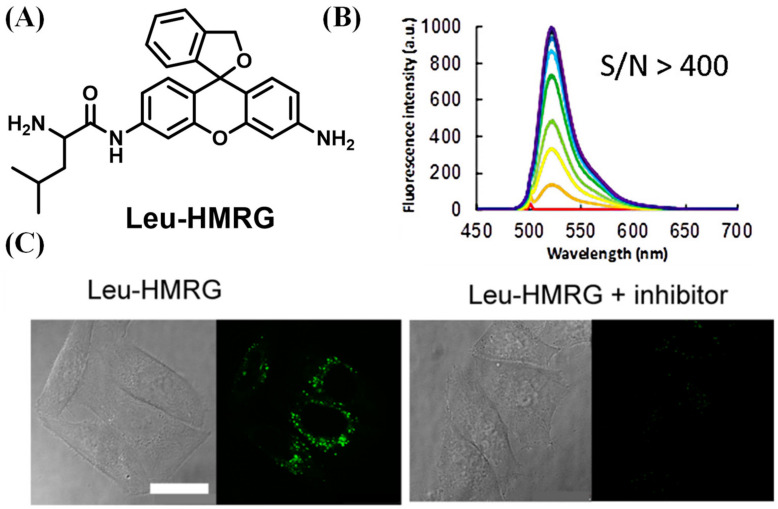
(**A**) Structure of Leu-HMRG. (**B**) Fluorescence response toward LAP. The reaction time was ranged from 0 (red line) to 30 min (purple line) (**C**) HEK 293 Cells imaging with/without inhibitor. (Reproduced with permission from [29], Copyright 2013, American Chemical Society).

Rhodamine green is a traditional and well-known dye, which is usually used as the fluorescent unit in probes design due to its high fluorescence quantum yield and long excitation/emission. However, these probes generally needed two steps of enzymatic reaction to generate a fluorescence signal, as they possess two recognition sites. Urano et al. proposed a general spirocyclization design strategy by employing the hydroxymethyl rhodamine green (HMRG) scaffold, which bypassed the limitations of the Rhodamine-based probe [29]. Although HMRG exhibits intense emission at the open structure, it displays no fluorescence signal due to the formation of a closed spirocyclic structure when one of the amino groups is acetylated (Ac-HMRG). Based on the character of HMRG, Urano’s group synthesized the LAP sensor, Leu-HMRG, which had a primary amine on one side of HMRG and an amide on another side of HMRG (Figure 3A). Leu-HMRG initially showed non-fluorescence at physiological pH, but the fluorescence intensity dramatically increased (more than 400-fold) upon treatment with LAP. It displayed dose dependence linearly related to the concentration of LAP (Figure 3B). Furthermore, the fluorescence of Leu-HMRG could not be activated by treatment with trypsin, chymotrypsin and cathepsin B, respectively. Its Kcat/Km value is four-fold higher than that of commercial LAP substrates, Leu-MCA and Leu-pNA in the detection of kinetic parameters. The in vitro biological experiment revealed an obvious luminescence signal consistent with the emission spectra of HMRG, which was obtained in HeLa cells, while the luminescence enhancement was blocked by adding bestatin (Figure 3C).

**Figure 4:**
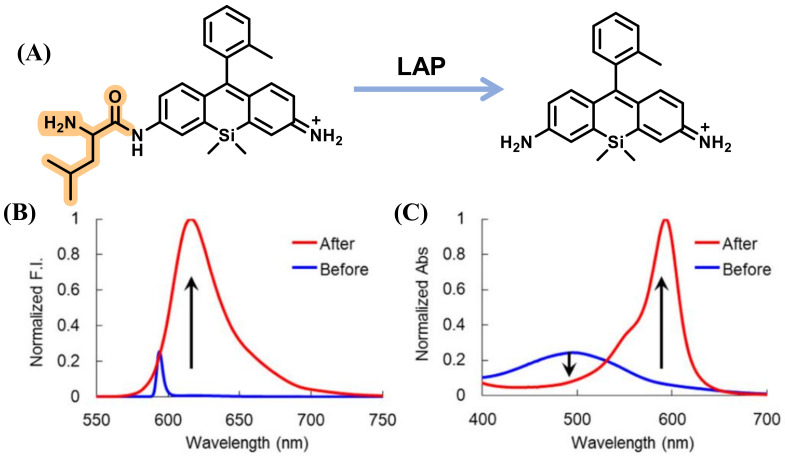
Reaction scheme of Leu-SiR600 with LAP (**A**). Fluorescence (**B**) and absorption (**C**) spectra of Leu-SiR600. (Reproduced with permission from [30], Copyright 2013, Elsevier).

**Figure 5:**
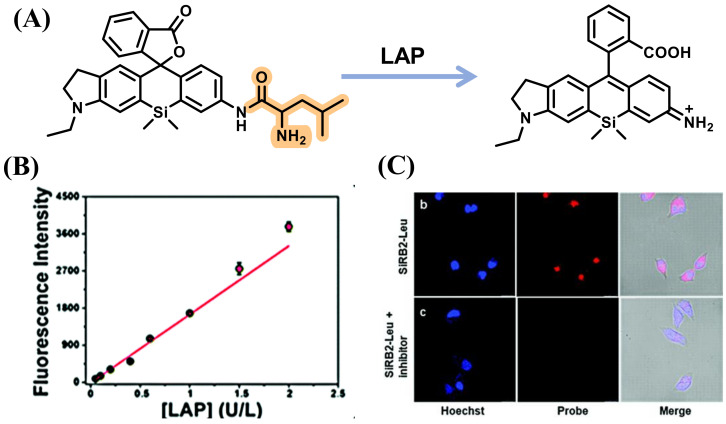
(**A**) Illustration of reaction of SiRB2-Leu and LAP. (**B**) Fluorescence response of SiRB2-Leu to LAP. (**C**) Cell imaging. (Reproduced with permission from [31], Copyright 2020, Royal Society of Chemistry).

Utilizing a similar dye, Yoshida et al. synthesized a novel Rhodamine Green analog, 2Me SiR600, by replacing the O atom with a Si atom at the 10 position of the xanthene moiety of Rhodamine Green. Then, 2Me SiR600 connected with L-leucine to construct the LAP probe, Leu-SiR600, which showed a strong fluorescence enhancement (151-fold) upon addition to LAP (Figure 4) [30]. Further, Cui et al. described an asymmetric carboxylated Si-rhodamine dye SiRB2-Leu that possessed durable tolerance for pH to detect the activity of LAP in Hoechst 33,342 cells successfully by incorporating L-leucine into the fluorescence group (Figure 5) [31].

**Figure 6:**
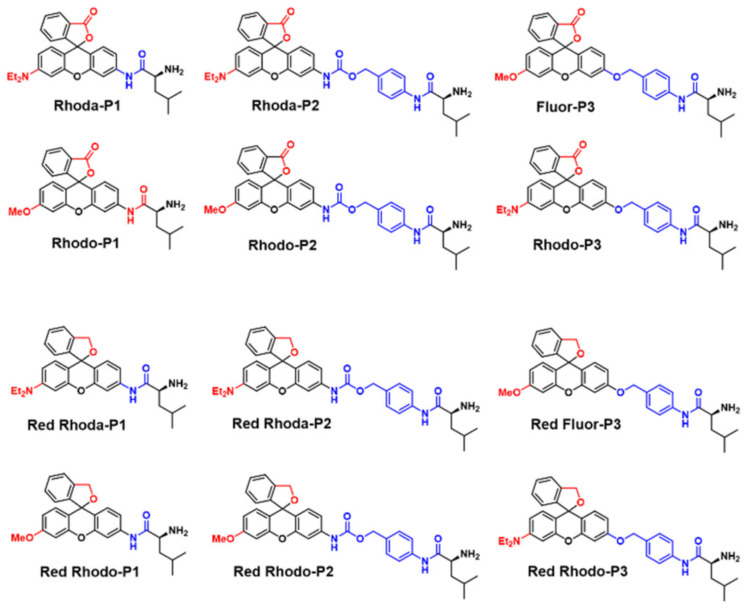
Chemical structure of LAP-responsive fluorescent probes. (Reproduced with permission from [32], Copyright 2022, Royal Society of Chemistry).

Chang et al. proved that the fluorescent probe can be utilized to exploit cleavable linkers for ligand-targeted drugs (LTDs) by modifying the LAP probe with various Rhodamine analogue and p-aminobenzyl carbamate (PABC) linkages (Figure 6) [32].

#### 2.1.3. Chemiluminescence LAP Probes

**Figure 7:**
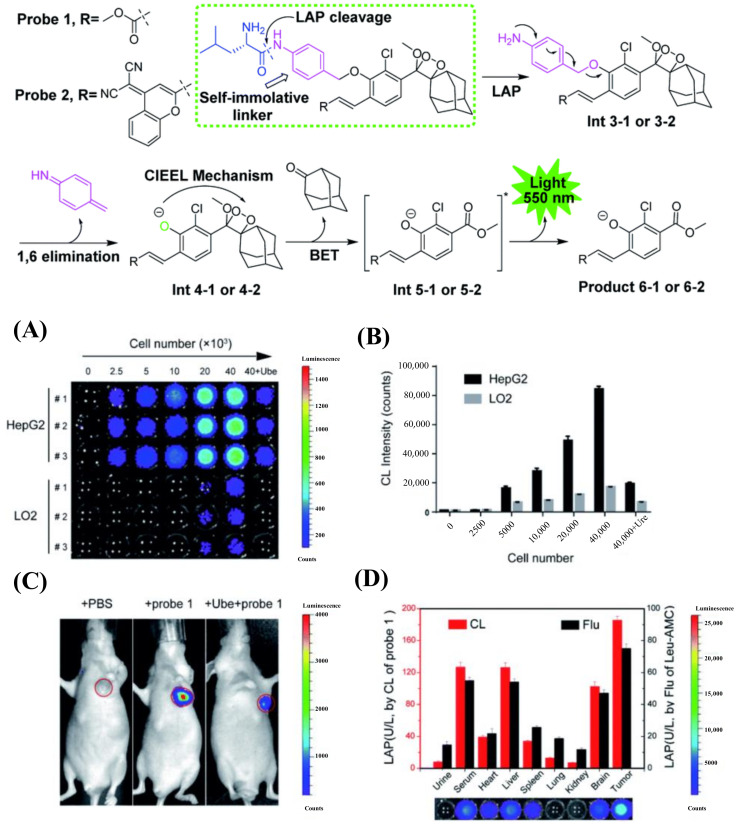
Reaction of probes 1 and 2 with LAP (**upper**). (**A**) Chemiluminescence images of HepG2 and LO2 cells. (**B**) Quantification of (**A**). Data. (**C**) Chemiluminescence imaging of mice. (**D**) Quantification of LAP level. (Reproduced with permission from [36], Copyright 2022, Royal Society of Chemistry).

Chemiluminescence is now a novel and powerful tool that has attracted considerable attention from researchers due to its generation of light via chemical reactions to provide chemiexcitation rather than via external optical excitation. Chemiluminescence can eliminate the ultrahigh background noise from biological tissue exhibited in the procedure of bioimaging compared with traditional fluorescence imaging [33,34,35]. Recently, Cheng’s team reported a chemiluminescent sensor for imaging LAP [36]. The probe is composed of an acryl-substituted phenoxy 1,2-dioxetane luminophore, a *p*-aminobenzyl alcohol is used as the self-immolative unit and LAP reaction substrate, which can be cleaved by LAP, leading to the release of chemiluminescence at 550 nm. When probe 1 was incubated with cells, it was able to differentiate the normal cells and tumor cells by testing the activity of LAP (Figure 7A,B). Probe 1 was then injected into the tumor-bearing mice to detect the endogenous LAP activity in living tumors (Figure 7C). Moreover, probe 1 was incubated with human tissue samples to distinguish liver cancer and normal tissue (Figure 7D).

#### 2.1.4. LAP Probes with AIEgens

**Figure 8:**
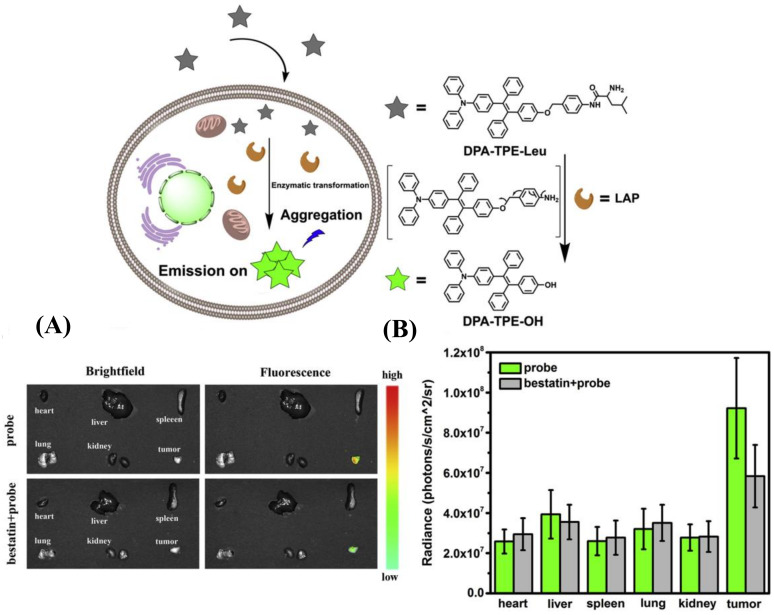
Recognition mechanism of DPA-TPE-Leu toward LAP (**upper**). (**A**) Bright field and fluorescent images of tumors and main organs with or without bestatin and (**B**) the relative intensity of (**A**). (Reproduced with permission from [44], Copyright 2018, Elsevier).

**Figure 9:**
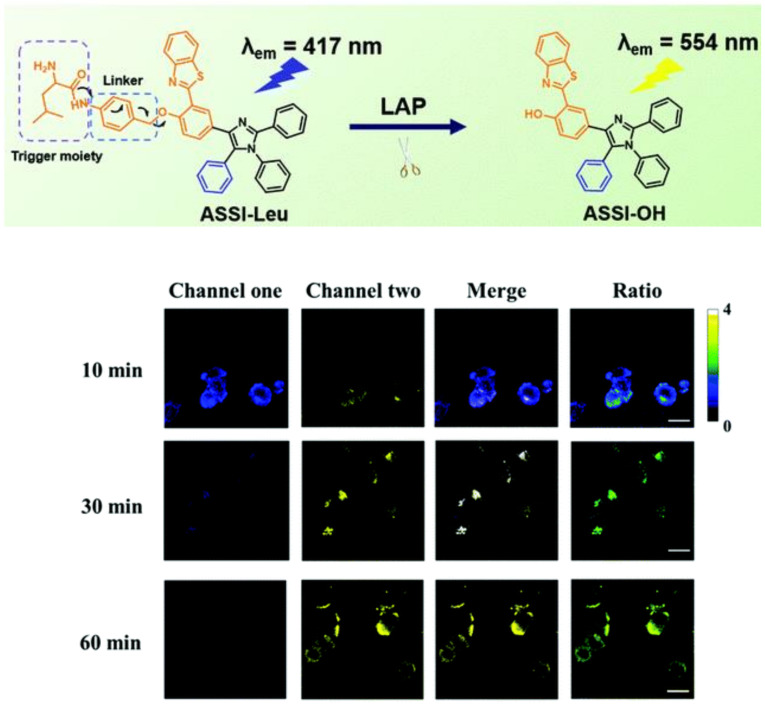
Proposed mechanism of LAP detection by ASSI-Leu (**upper**) and HepG2 cells imaging (**below**). (Reproduced with permission from [45], Copyright 2021, Royal Society of Chemistry).

Unlike traditional dye, aggregation-induced emission (AIE) fluorophores emit strong fluorescence upon aggregation rather than quenching fluorescence due to the inhibition of the non-radiative decay via the restriction of intramolecular rotation/vibration [37,38,39,40,41,42,43]. This feature granted AIEgens high resistance to photobleaching, as well as enhanced sensitivity. Wu et al. employed tetraphenylethylene (TPE) with the connection of diphenylamine as the fluorescence reporter and L-leucine amide as the recognition site to develop the AIE luminescent probe for LAP, DPA-TPE-Leu [44]. The probe was successfully applied to detect the LAP activity in HepG2 cells and the tumor tissue of tumor-bearing mice inoculated with HepG2 cells (Figure 8). After that, Zeng and coworkers devised an innovative method that integrated the 2-(2′-hydroxy-phenyl) benzothiazole (HBT) skeleton into the five-position aromatic ring to synthesize a fluorescence indicator with asymmetric substituents at the 4,5-positions [45]. Connecting the above fluorophore with the LAP recognition substrate, ASSI-Leu was built and performed to effectively image the LAP vigor of HepG2 cells and zebrafish (Figure 9). 

#### 2.1.5. Multi-Detection Probes for Imaging LAP and Diseases Diagnosis

**Figure 10:**
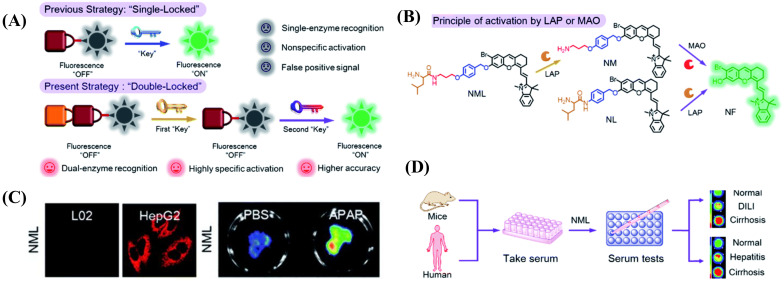
(**A**) Design strategy and (**B**) response mechanism of NML. NML differentiated hepatoma cells from normal cells (**C**) and distinguished hepatopathy in mouse or human serum (**D**). (Reproduced with permission from [50], Copyright 2019, Royal Society of Chemistry).

The development of disease is a fairly complicated procedure involving a range of physiological processes, such as abnormal levels of biomolecules, deviation of the pH value, redox system imbalance, etc. [46,47,48,49]. Probes with only a single trigger site, however, are difficult to use for precise diagnoses, as a false positive signal might also be recorded in a healthy area. To overcome this issue, Zhang et al. engineered a “double-locked” molecular probe, NML, by binding a leucine (first “lock” for LAP) to a propylamine group via a pseudo-peptide bond (second “lock” for MAO). It was then connected through an ether bond to the fluorescence reporter by employing p-hydroxybenzyl as the elimination type, which will enhance the steric hindrance for MAO recognition to increase the detection limit (Figure 10A) [50]. When both LAP and MAO were exposed to the probe, the fluorescence signal at 720 nm showed an obvious increase; otherwise, NML remained in the turn-off state (Figure 10B). In cell experiments, NML showed an obvious fluorescence signal in HepG2 cells, while it displayed nearly no fluorescence emission in the LO2 cells and the HepG2 cells treated with the inhibitors (bestatin and clorgiline) of the two enzymes (Figure 10C). Using acetaminophen (APAP) to build a drug-induced liver injury model, pronounced differentiation between normal and injured liver in fluorescence images was observed after injection with NML. Significantly, the changed fluorescence intensity of NML was capable of distinguishing different models of mouse serum, including cirrhotic serum (3.3 ± 0.15 folds enhancement), DILI serum (1.8 ± 0.12 folds enhancement), and normal serum, thus identifying different hepatopathies (Figure 10D). 

**Figure 11:**
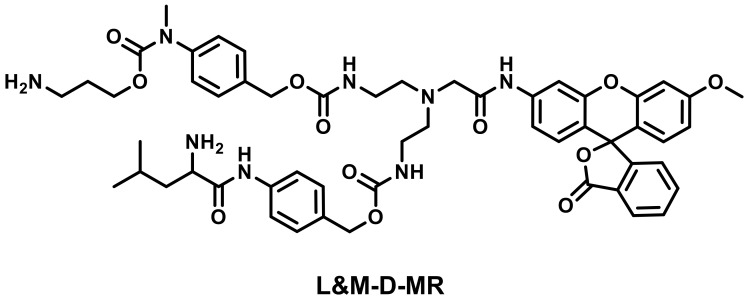
Chemical structure of L&M-D-MR.

In a similar fashion, Liu et al. designed a dien-based AND logic probe, namely L&M-D-MR, that consisted of a LAP-responsive group and an MAO-responsive unit and could be prepared as the test strip for the detection of LAP and MAO [51]. In addition, L&M-D-MR also has the ability to identify different kinds of hepatopathy (Figure 11).

**Figure 12:**
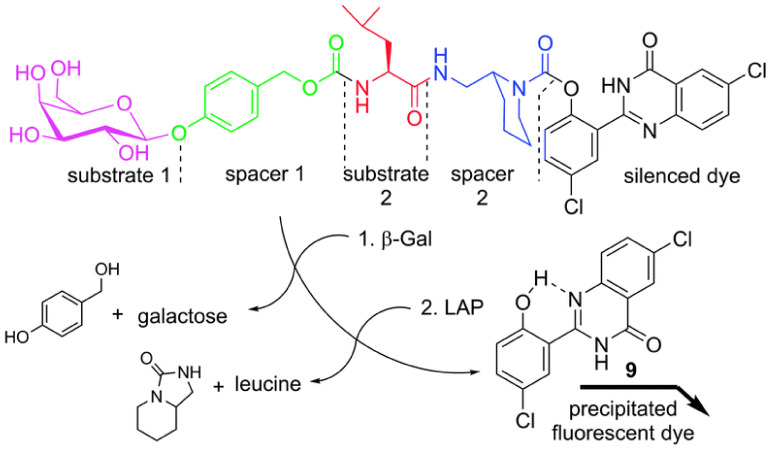
Proposing sensing mechanism of probe 1. (Reproduced with permission from [52], Copyright 2014, Royal Society of Chemistry).

Hasserodt et al. presented an AND logic gate probe that needed to undergo four successive chemical reactions to turn on the fluorescence for imaging β-gal and LAP (Figure 12) [52].

**Figure 13:**
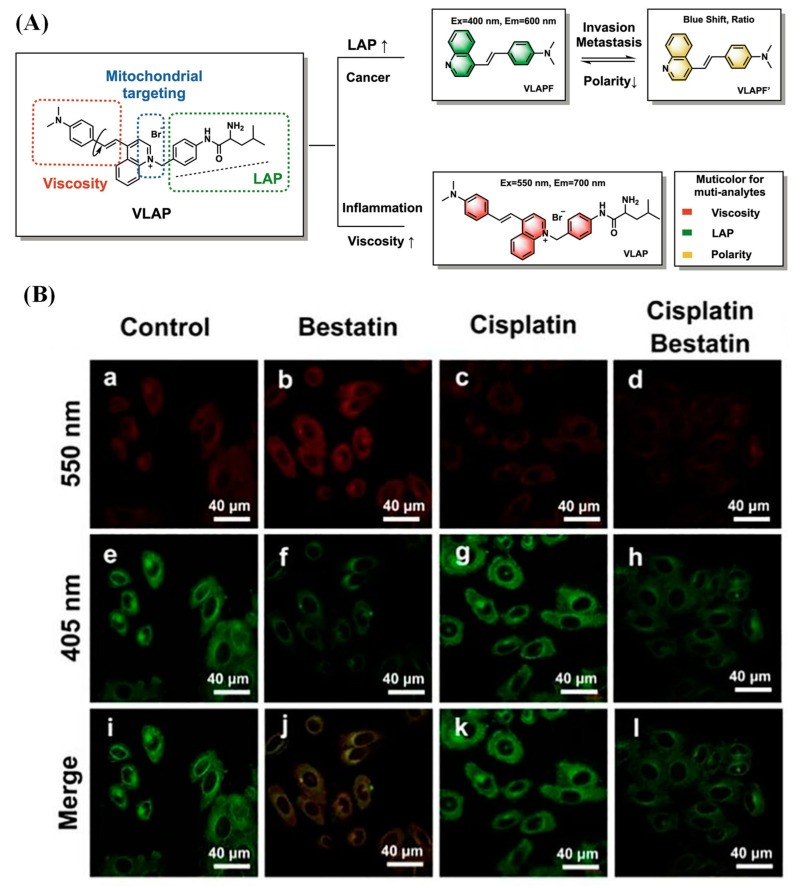
(**A**) Design principle of VLAP. (**B**) Hep G2 cells imaging with different LAP inhibitor. (Reproduced with permission from [53], Copyright 2022, Elsevier).

Recently, Li et al. reported a fluorescent probe that could monitor the relevant proteins for the development of inflammation–cancer and image the cell status through simultaneous multicolor detection of three analytes (LAP, viscosity and polarity) (Figure 13) [53].

#### 2.1.6. LAP Probes with Targeting Capability

**Figure 14:**
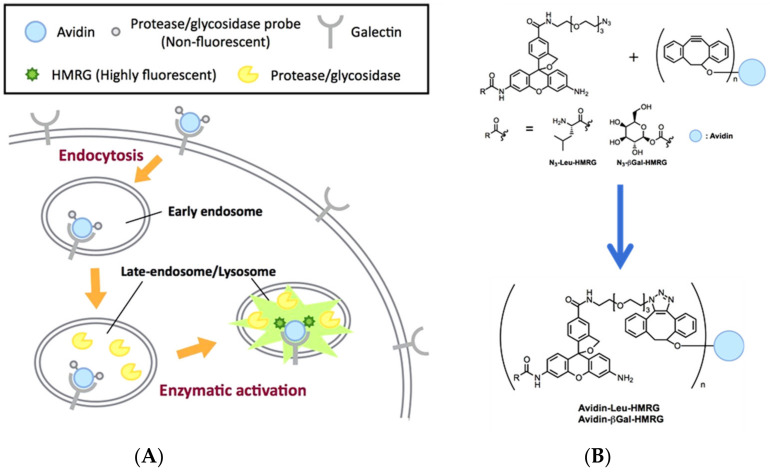
The activation mechanism (**A**) and structure (**B**) of avidin–protease probe conjugates. (Reproduced with permission from [59], Copyright 2019, Elsevier).

In addition to specificity, sensitivity, real-time feedback, etc., the accurate localization of the lesion site for fluorescence imaging is also an important evaluation criterion of the probe, which is necessary for practical application [54,55,56,57,58]. Utilizing click chemistry, Urano and coworker prepared Avidin-Leu-HMRG by combining N_3_-Leu-HMRG decorated with azide moiety and the avidin pre-labeled with cyclooctyne-NHS. The use of Avidin-Leu-HMRG was valid for effectively targeting the tumor site for LAP detection (Figure 14) [59].

**Figure 15:**
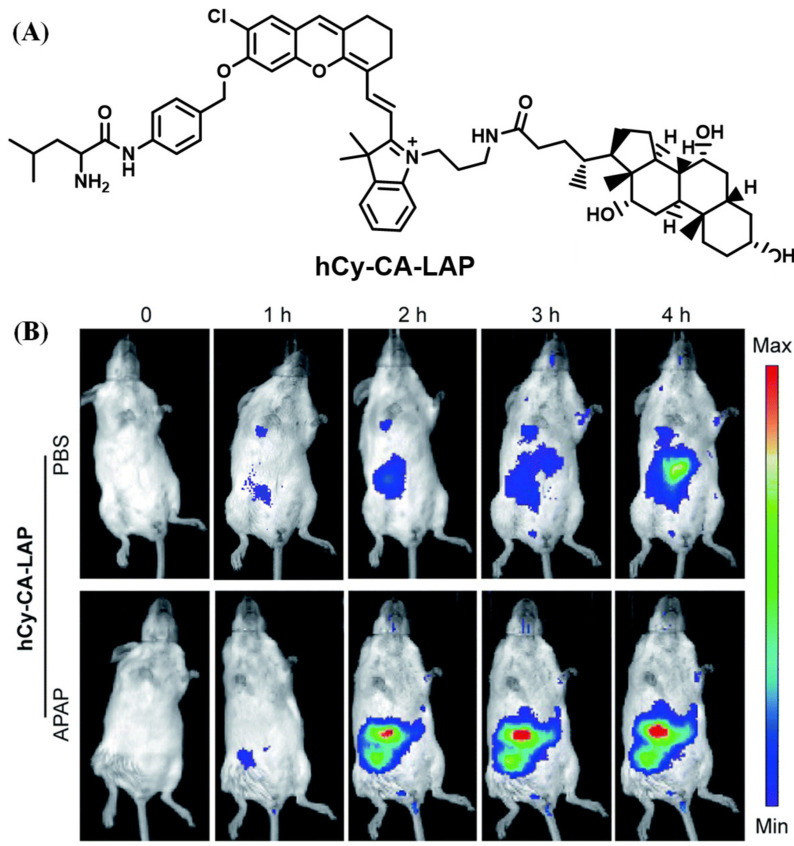
(**A**) Chemical structure of hCy-CA-LAP. (**B**) Time-dependent in vivo imaging of mice. (Reproduced with permission from [60], Copyright 2021, Royal Society of Chemistry).

**Figure 16:**
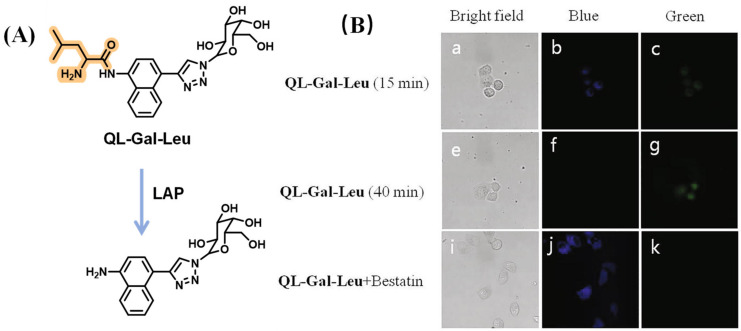
(**A**) Illustration of reaction of QL-Gal-Leu and LAP. (**B**) Cell imaging. (Reproduced with permission from [61], Copyright 2021, Elsevier).

Su’s group developed a LAP fluorogenic sensor that decorated with cholic acid to greatly improve the targeting ability of the intrahepatic. It was employed to accurately image the acute liver injury and cholestatic liver injury induced by APAP and rifampicin (RFP), respectively (Figure 15) [60]. Furthermore, an 8-aminoquinoline fluorescent moiety modified with a tumor-targeted unit as well as galactose and L-leucine was proposed by Shen’s team and applied to detect the LAP in HepG2 cells (Figure 16) [61].

#### 2.1.7. Fluorescent LAP Probes for Integrated Diagnosis and Treatment

**Figure 17:**
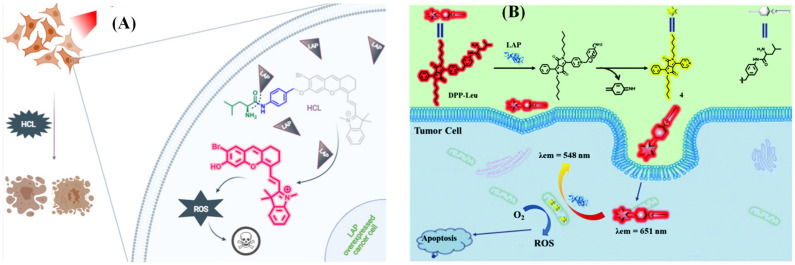
Illustration of activation mechanism of HCL (**A**), reproduced with permission from [69], Copyright 2021, Elsevier) and DPP-Leu ((**B**), reproduced with permission from [72], Copyright 2019, Royal Society of Chemistry).

**Figure 18:**
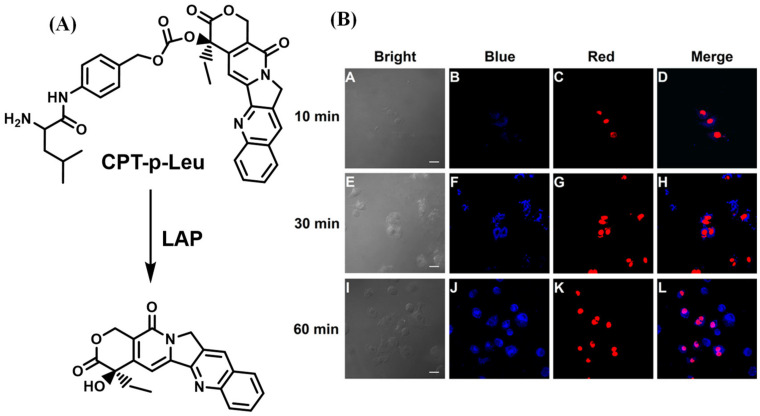
(**A**) Schematic illustration of response mechanism to CPT-p-Leu to LAP. (**B**) Fluorescence images of A549 cells that treated with CPT-p-Leu. (reproduced with permission from [73], Copyright 2019, American Chemical Society).

The small molecular fluorescent probe is not only capable of conducting biological imaging but can also be designed as a functional agent to treat the disease effectively [62,63,64,65,66,67,68,69,70]. Kolemen’s group devised the photosensitizer HCL by decorating hemicyanine with heavy atom bromine (Figure 17A), and Hua et al. proposed a ratiometric fluorescent probe DPP-Leu based on diketopyrrolopyrrole (DPP) (Figure 17B) to perform photodynamic therapy efficaciously for cancer cells overexpressing LAP [71,72]. Meanwhile, He and coworkers reported a theragnostic prodrug CPT-Leu that utilizes camptothecin as the fluorescent indicator and drug, which was capable of conducting the chemotherapy toward the LAP overexpression in cancer cells (Figure 18) [73].

#### 2.1.8. NIR Probes for Detecting LAP

**Figure 19:**
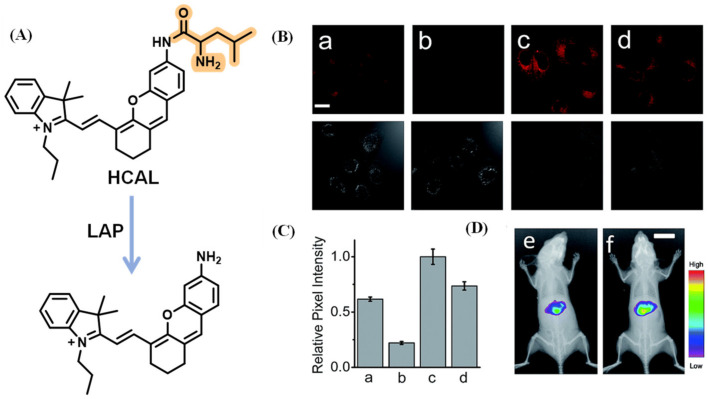
(**A**) Sensing mechanism of HCAL for LAP. (**B**) Confocal images of the LO2 cells ((a) control, (b) added bestatin, (c) added Ace, (d) added Ace and acetylcysteine) and (**C**) quantification of fluorescence. (**D**) In vivo fluorescence imaging of mice preinjected with (**e**) PBS and (**f**) HCAL. (Reproduced with permission from [74], Copyright 2017, Royal Society of Chemistry).

**Figure 20:**
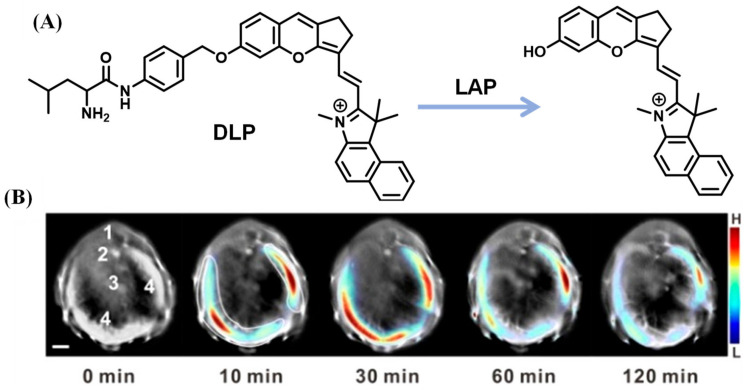
(**A**) Chemical structure and recognition mechanism of DLP. (**B**) Time-dependent cross-sectional MSOT images (1. spinal cord; 2. aorta; 3. venacava; 4. liver). (Reproduced with permission from [75], Copyright 2019, American Chemical Society).

**Figure 21:**
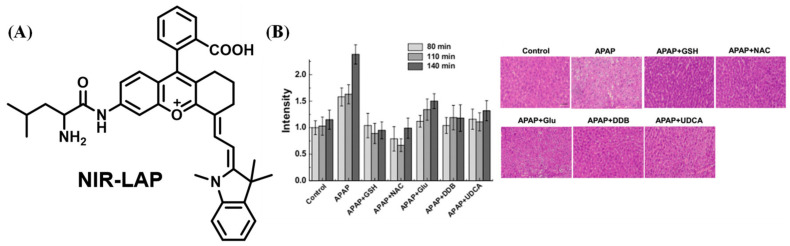
(**A**) Structure of NIR-LAP. (**B**) Investigation of medicine by NIR-LAP. (Reproduced with permission from [76], Copyright 2019, American Chemical Society).

Fluorescent probes with near-infrared (NIR) emission, which serve as powerful bioimaging tools, were favored by researchers due to their deep tissue penetration and lower background autofluorescence. Employing hemicyanine, a typical NIR fluorophore, Ma et al. designed the probe, HCAL, which revealed that increased LAP may be relative to the deficiency in biothiols (Figure 19) [74]. With a similar structure to hemicyanine, a LAP probe, DLP, was synthesized by Wu et al. and applied to detect the LAP increase and locate the foci of liver injury through multispectral optoacoustic tomography (MSOT) (Figure 20) [75]. Yuan’s lab reported that the probe NIR-LAP consisted of (E)-6-amino-9-(2-carboxyphenyl)-4-(2-((Z)-1,3,3-trimethylindolin-2-ylidene)ethylidene)-1,2,3,4-tetrahydroxanthylium and L-leucine (Figure 21), which was capable of evaluating the liver protective effects of hepatoprotective medicine [76].

**Figure 22:**
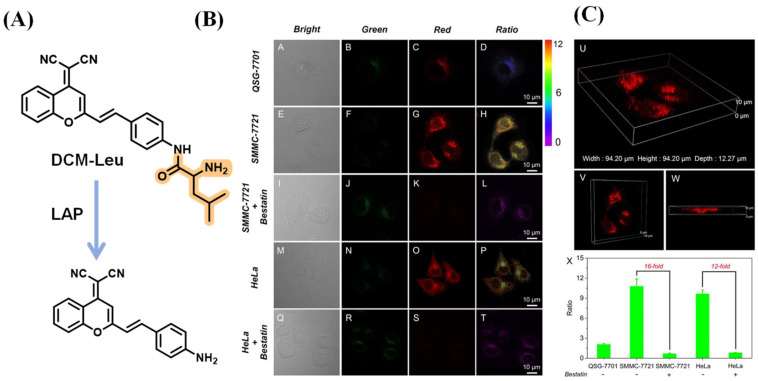
(**A**) Illustration of sensing mechanism of DCM-Leu. (**B**) Cells images. (**C**) Three-dimensional CLSM images and LAP activity in different cells with or without bestatin. (Reproduced with permission from [77], Copyright 2016, American Chemical Society).

Zhu et al. incorporated the L-leucine unit as the fluorescence quencher and recognition moiety into the dicyanomethylene-4H-pyran (DCM) group to construct the LAP probe, DCM-Leu. As the cell imaging illustrated, the luminescence signal recorded in SMMC-7721 cells was more intense than that in QSG-7701, while the fluorescence emission of the two kinds of cells was blocked by adding bestatin. Through the different views of three-dimensional confocal imaging, the distribution of intracellular LAP can be observed; they are mainly located in the cytoplasm (Figure 22) [77].

**Figure 23:**
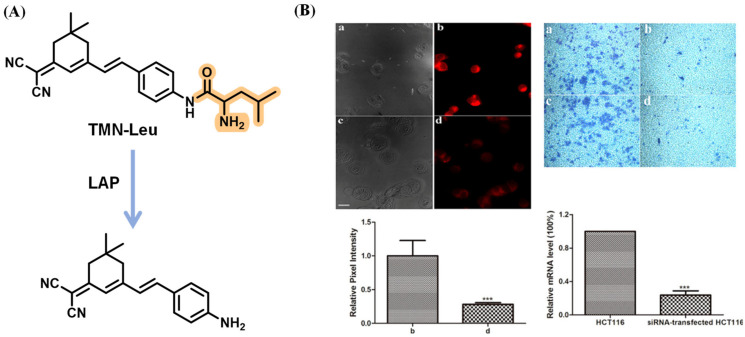
(**A**) Proposed sensing mechanism of TMN-Leu for LAP enzymatic activation. (**B**) HCT116 cell imaging and their relative invasion activity, *** *p* < 0.001. (Reproduced with permission from [78], Copyright 2017, American Chemical Society).

**Figure 24:**
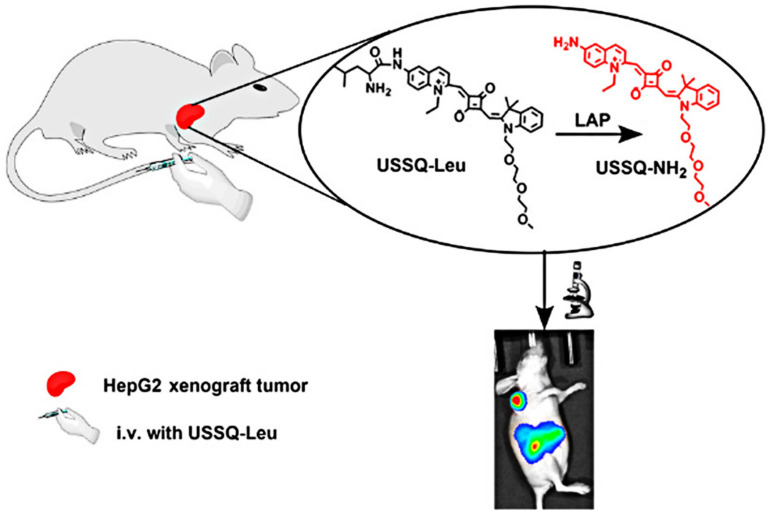
Proposing sensing mechanism of USSQ-Leu. (Reproduced with permission from [79], Copyright 2018, American Chemical Society).

**Figure 25:**
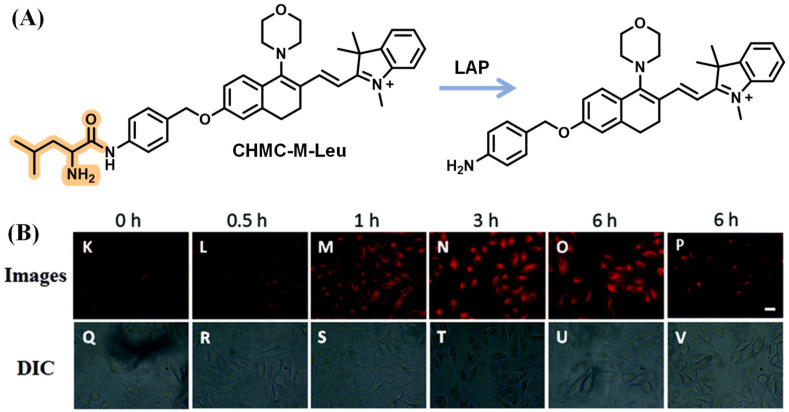
(**A**) Structure of CHMC-M-Leu. (**B**) Time-dependent images of HeLa cells. (Reproduced with permission from [80], Copyright 2019, Royal Society of Chemistry).

After that, Kong’s lab synthesized the mitochondria-targeting fluorescent probe TMN-Leu to monitor the LAP by employing the dicyanoisophorone chromophore similar to DCM. It was revealed that the cells with higher LAP activity were more invasive, implying that LAP might serve as an indicator reflecting the intrinsic invasion capability of cancer cells (Figure 23) [78]. Through decorating squaraine dye with the OEG (triethylene glycol) group to improve aqueous solubility, the USSQ-Leu probe reported by Wu et al. was successfully applied to monitor endogenous LAP both in vitro and in vivo (Figure 24) [79]. In 2019, Wang et al. proposed a water-soluble fluorescent probe, CHMC-M-Leu, which effectively tracked endogenous LAP activity in living cells (Figure 25) [80].

**Figure 26:**
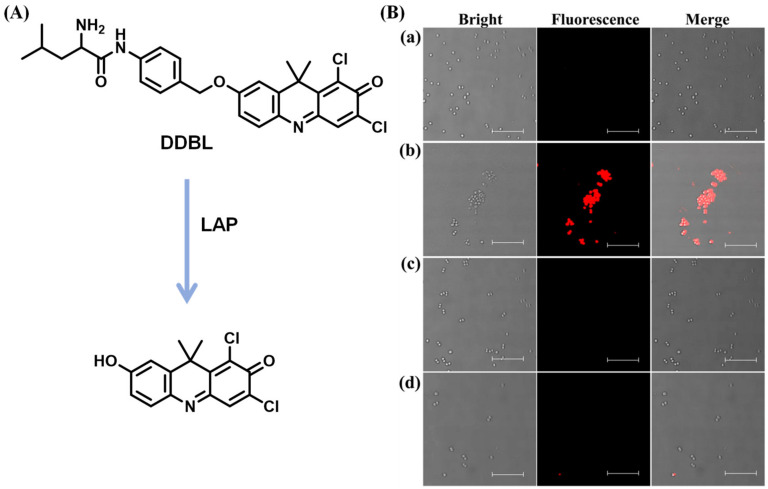
(**A**) Illustration of the enzymatic hydrolysis of DDBL mediated by LAP. (**B**) Fluorescence imaging of *S. aureus* with different treatment: (a) blank, (b) DDBL, (c) DDBL and AKBA, (d) DDBL and carnosic acid (scale bars are 10 µm). (Reproduced with permission from [85], Copyright 2021, American Chemical Society).

LAP generated in bacteria served as a vital virulence factor associated with the formation of bacterial biofilm and the pathogenicity of pathogens, which cause acute or chronic diseases [81,82,83,84]. Recently, a novel NIR fluorescent probe DDBL composed of the chromophore 7-hydroxy-9H (1,3-dichloro-9,9-dimethylacridin-2-one) (DDAO) and reaction unit L-leucine used to track bacterial LAP was developed by Wang et al. [85] After coincubation with intestinal bacteria, the fluorescence emission of DDAO was observed, suggesting that the generation of LAP occurs in bacterial strains. In addition, DDBL was capable of screening inhibitors of LAP for *S*. *aureus*, revealing that 3-acetyl-11-keto-β-boswellic acid (AKBA) has the ability to decrease the pathogenicity of infectious diseases (sepsis, etc.) for *S*. *aureus* (Figure 26).

**Figure 27:**
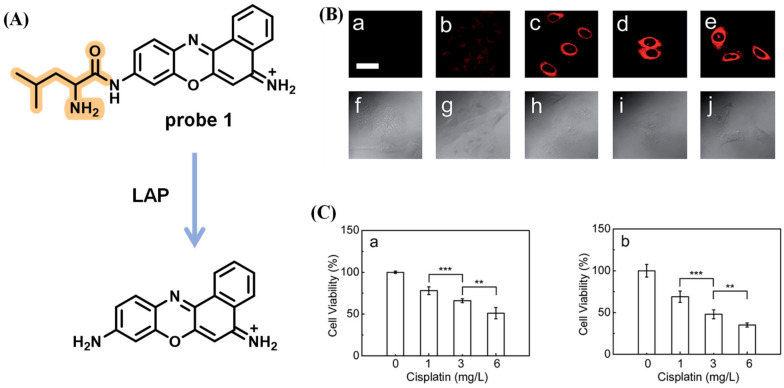
(**A**) Reaction of probe 1 with LAP. (**B**) Imaging of HepG2 cells treated with various concentration of cisplatin. (**C**) MTT assay (*** *p* < 0.001, ** *p* < 0.01). (Reproduced with permission from [88], Copyright 2016, Royal Society of Chemistry).

Cancer is one of the diseases with the highest mortality rate worldwide, and there are a lot of obstacles during treatment, such as intrinsic or acquired resistance and tumor immune escape [86,87]. To investigate whether LAP affects tumor therapy using cisplatin, a classical anticancer drug, an ultra-sensitive fluorescent sensor for LAP was reported by Ma et al. (Figure 27A) [88]. In bioimaging, the relative pixel intensity in HepG2 cells is about two times higher than that in A549 cells, implying that HepG2 cells possess a higher concentration of intracellular LAP. The luminescence signal of probe 1 significantly decreased when pretreated with ubenimex, an inhibitor of LAP. Next, the effects of cisplatin treatment with different concentrations (0–2 mg/L) and incubation times were studied. The results showed that the intracellular LAP activity/concentration gradually enhanced upon the addition of a concentration range of 0–1 mg/L of cisplatin or reached a maximum after 6 h incubation with cisplatin, respectively (Figure 27B). Furthermore, the experiment recorded the activity of HepG2 and A549 cells via MTT assay. The results revealed that the viability of both cells was gradually reduced with the increase in cisplatin from 1 to 6 mg/L. HepG2 cells, with a higher concentration of endogenous LAP have greater resistance than A549 cells, which indicates that the tumor cells with a higher concentration of intracellular LAP may obtain more significant intrinsic resistance toward cisplatin via the alteration of the intracellular interactive pathways (Figure 27C).

#### 2.1.9. Other Types of Leu Residue Hydrolysis Probes

**Figure 28:**
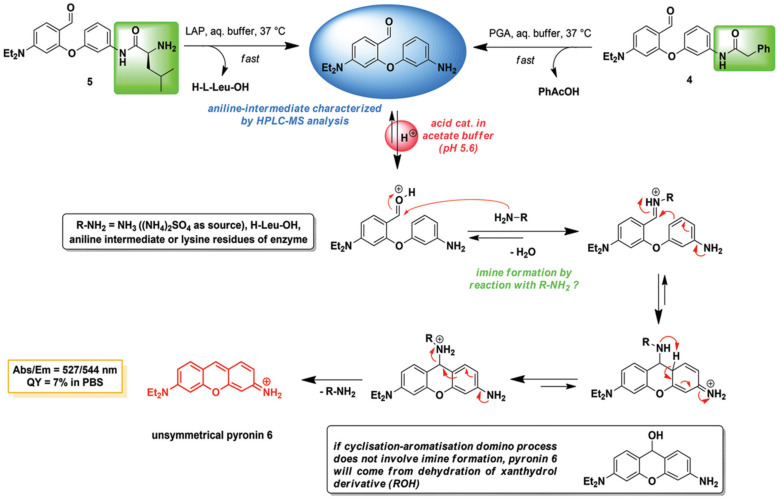
Schematic illustration for detection LAP by the “covalent-assembly” principle. (Reproduced with permission from [90], Copyright 2017, Royal Society of Chemistry).

To extend the performance of the probe designed using the “covalent-assembly” [89] principle for enzymes imaging and dual- or multi-analyte detection, Romieu’s team proposed the in situ formation of pyronin dyes triggered using enzymatic species (LAP or penicillin Gacylase (PGA)) (Figure 28) [90].

**Figure 29:**
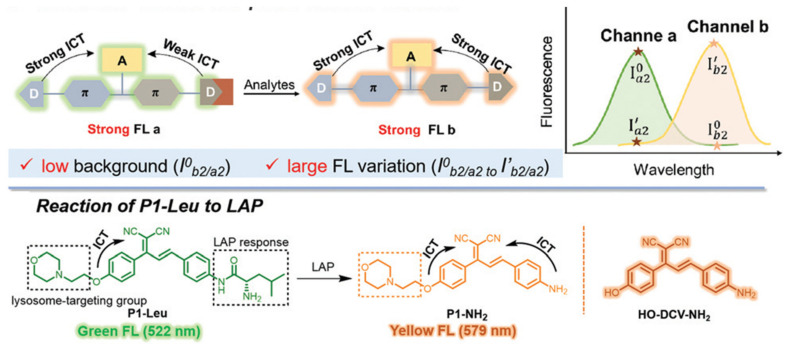
Design principle and response mechanism of P1-Leu. (Reproduced with permission from [91], Copyright 2022, Royal Society of Chemistry).

**Figure 30:**
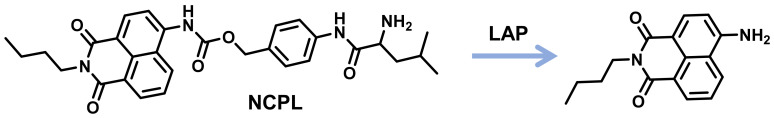
Schematic illustration of NCPL reaction with LAP. (Reproduced with permission from [92], Copyright 2021, Elsevier).

Lately, a lysosome-targeted ratiometric probe based on the D-A-D structure that showed a low ratiometric background and large fluorescence variation was developed by Wang et al. to distinguish LAP concentration in different cells (Figure 29) [91]. Cui et al. designed the LAP probe NCPL with 4-amino1,8-naphthalimide dye to image the DILI (Figure 30) [92].

### 2.2. Intramolecular Ring Formation

**Figure 31:**
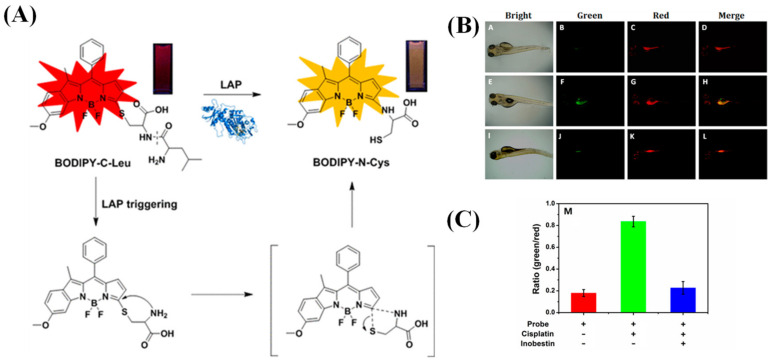
(**A**) Illustration of fluorescence change of BODIPY-Cys triggered by LAP. (**B**) Fluorescent images for zebrafish larvae with different treatment: (A–D) BODIPY-C-Leu, (E–H) BODIPY-C-Leu and cisplatin, (I–L) BODIPY-C-Leu and cisplatin and inobestin. (**C**) Ratio from green channel to red channel of (**B**). (Reproduced with permission from [93], Copyright 2017, American Chemical Society).

A novel ratio fluorescent probe, BODIPY-C-Leu, with an asymmetrical BODIPY as the luminescence emitter and a dipeptide (Cys-Leu) with free thiol function as the reaction unit, was reported by Wang et al. [93]. The wavelength of the absorption and emission of BODIPY-C-Leu shifted from 578 and 601 nm to 505 and 578 nm, respectively, triggered by LAP. This can be attributed to the cleavage of the amide bond in dipeptide and the further released free amino group, followed by an intramolecular displacement of thiolate to yield an amino-substituted BODIPY-Cys adduct via a five-membered cyclic transition state to realize the S→N conversion (Figure 31A). In biological tests, the activity of LAP in HeLa cells and zebrafish was monitored efficaciously by BODIPY-C-Leu (Figure 31B,C). 

**Figure 32:**
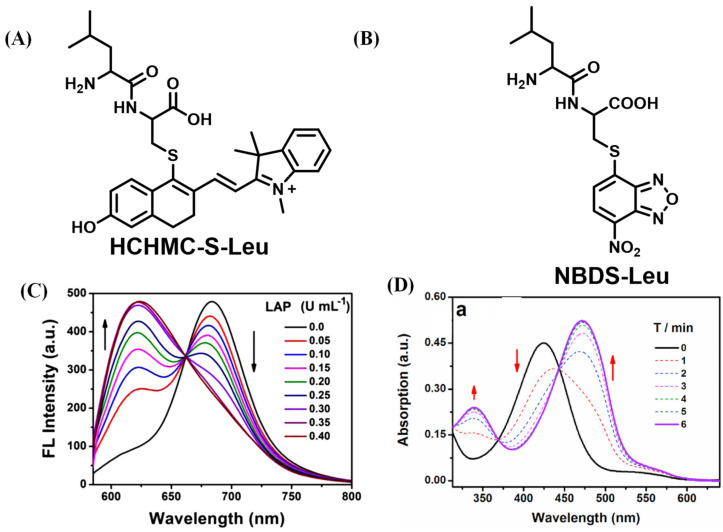
Structure of HCHMC-S-Leu (**A**) and NBDS-Leu (**B**). Fluorescence response of HCHMC-S-Leu ((**C**), Reproduced with permission from [94], Copyright 2020, Elsevier) and NBDS-Leu ((**D**), Reproduced with permission from [95], Copyright 2021, Elsevier) to LAP.

**Figure 33:**
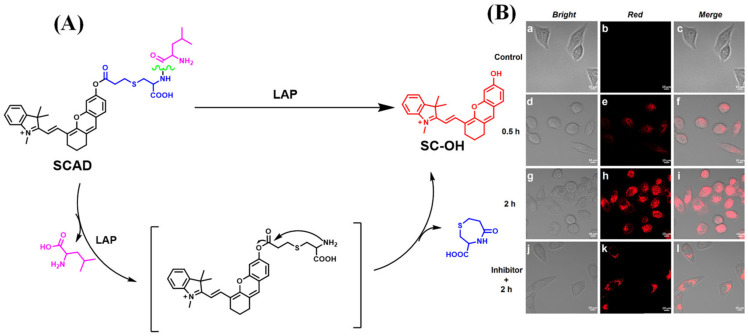
(**A**) Response mechanism of CY-P toward LAP. (**B**) Fluorescence imaging of HeLa cells incubated with CY-P for different time. (Reproduced with permission from [96], Copyright 2021, Elsevier).

After this work, Wang’s team continued to report on another two probes, attaching the same reaction rearrangement unit to chloro-hydroxyl-merocyanine (CHMC) (Figure 32A) and 4-chloro-7-nitro-2,1,3-benzoxadiazole (NBD-Cl) fluorescent scaffold (Figure 32B) [94,95]. In further research, Wang and teammates grafted a dipeptide into a semicyanine skeleton through an acryloyl to construct the probe that underwent intramolecular cyclization to generate fluorescence output after the reaction with LAP (Figure 33) [96].

## 3. Conclusions and Outlook

In this review, the design principle of the majority of the LAP probes is based on the formation of primary amines via the hydrolyzation of leu residues with LAP, causing the fluorescence change of fluorophore. While the other probes are based on the transition state of the five-membered ring to realize S→N conversion after the reaction with LAP led to the fluorescence change. These probes are beneficial, as they illustrate the distribution and intracellular movement of the LAP; this may shed light on the role of the LAP in various diseases and pathological processes, such as cancer and drug-induced liver injury. 

The development of fluorescent probes and chemosensors has blossomed in recent decades, especially reaction-based probes known as chemodosimeters. These advances have been accompanied by the development of fluorescent probes for enzymes. Although the LAP sensor has made remarkable progress, there are still many challenges and opportunities: (1) Development of probes of the NIR-II platform is necessary due to the high tissue penetration and penetration depth of the NIR-II. (2) It is of great significance to develop a LAP probe with long-term tracking and high stability to monitor diseases and physiological processes. (3) Combining fluorescence imaging with other imaging methods (such as MRI, PET, CT, etc.), multi-mode imaging can be performed to improve the accuracy of diagnosis. We hope that the design strategies of these LAP probes can provide guidance for the subsequent development of LAP or other types of probes. At the same time, we hope these probes will help patients in the clinic.

## Data Availability

Not available.
